# Body surface rewarming in fully and partially hypothermic king penguins

**DOI:** 10.1007/s00360-020-01294-1

**Published:** 2020-07-12

**Authors:** Agnès Lewden, Andreas Nord, Batshéva Bonnet, Florent Chauvet, André Ancel, Dominic J. McCafferty

**Affiliations:** 1 Département Ecologie, Université de Strasbourg, CNRS, Physiologie et Ethologie, IPHC UMR 7178, 67000 Strasbourg, France; 2 School of Biomedical Sciences, Faculty of Biological Sciences, University of Leeds, Woodhouse Lane, Leeds, LS2 9JT UK; 3 Department of Biology, Section for Evolutionary Ecology, Lund University, 223 62 Lund, Sweden; 4 Scottish Centre for Ecology and the Natural Environment, Institute of Biodiversity, Animal Health and Comparative Medicine, College of Medical, Veterinary and Life Sciences, University of Glasgow, Rowardennan, Glasgow, G63 0AW Scotland UK; 5 Centre D’Etudes Biologiques de Chizé, CNRS, UMR 7372, 79360 Villiers en Bois, France

**Keywords:** Thermal windows, Heterothermy, Thermal imaging, Vasoconstriction, Vasodilation, Vasomotor response, Thermoregulation, Bird

## Abstract

Penguins face a major thermal transition when returning to land in a hypothermic state after a foraging trip. Uninsulated appendages (flippers and feet) could provide flexible heat exchange during subsequent rewarming. Here, we tested the hypothesis that peripheral vasodilation could be delayed during this recovery stage. To this end, we designed an experiment to examine patterns of surface rewarming in fully hypothermic (the cloaca and peripheral regions (here; flippers, feet and the breast) < 37 °C) and partially hypothermic (cloaca at normothermia ≥ 37 °C, but periphery at hypothermia) king penguins (*Aptenodytes patagonicus*) when they rewarmed in the laboratory. Both groups rewarmed during the 21 min observation period, but the temperature changes were larger in fully than in partially hypothermic birds. Moreover, we observed a 5 min delay of peripheral temperature in fully compared to partially hypothermic birds, suggesting that this process was impacted by low internal temperature. To investigate whether our laboratory data were applicable to field conditions, we also recorded surface temperatures of free-ranging penguins after they came ashore to the colony. Initial surface temperatures were lower in these birds compared to in those that rewarmed in the laboratory, and changed less over a comparable period of time on land. This could be explained both by environmental conditions and possible handling-induced thermogenesis in the laboratory. Nevertheless, this study demonstrated that appendage vasodilation is flexibly used during rewarming and that recovery may be influenced by both internal temperature and environmental conditions when penguins transition from sea to land.

**Electronic supplementary material:**

The online version of this article (10.1007/s00360-020-01294-1) contains supplementary material, which is available to authorized users.

## Introduction

Seabirds and pinnipeds spend a large part of their lives at sea but periodically come to land for reproduction, moult or rest (Feltz and Fay 1966; Ling 1970; Croxall 1982; Hammond et al. 1988; Watts 1992, 1996). The return to land is a major thermal transition, because air has physical characteristics (e.g., thermal conductivity) that substantially reduces the rate of heat loss compared to water (Bullard and Rapp 1970; Nadel 1984; Ponganis 2015) and air temperatures are often higher than sea temperatures.

To reduce the rate of heat loss at sea, the body trunk of marine endotherms is well insulated by dense pelage or a thick layer of subcutaneous fat. Furthermore, marine endotherms generally show local heterothermy in appendages and the body trunk (Culik et al. 1996; Bevan et al. 1997; Handrich et al. 1997; Boyd 2000) which not only extends dive duration by lowering tissue demands for oxygen (Scholander 1940) but also decreases the rate of heat loss by reducing the temperature gradient between the body and the environment. Reduced appendage temperature is achieved by massive peripheral vasoconstriction and countercurrent heat exchange in the appendages (Pabst et al. 1999; Williams and Worthy 2002). The latter is particularly important, because the appendages are relatively fat free, and at most insulated by short feathers or hair. Accordingly, heat loss from the appendages was estimated to 8–28% of total body heat loss in whales and seals (Ryg et al. 1993). However, vasoconstriction may reduce heat loss from the flippers to only 2–6% of total body heat loss in 0 °C water, compared to 19–48% without vasoconstriction at 24 °C (Kvadsheim and Folkow 1997).

In contrast to when they are submerged, marine endotherms typically maintain stable high body temperature on land. For example, dry king penguins (*Aptenodytes patgonicus*) on land have a core body temperature of 37 °C (Schmidt et al. 2006; Lewden et al. 2017a). In seals, vasodilation of the uninsulated appendages can rapidly increase heat loss during periodic haul-outs onto land. This has been proposed to help maintain core body temperature and avoid hyperthermia in the warmer and less conductive air (Watts 1992). For this reason, uninsulated body parts such as appendages have been characterized as ‘thermal windows’ that allow flexible heat transfer in a range of environments (e.g., Klir and Heath 1992; Williams 1990; Norris et al. 2010; Erdsack et al. 2012; Tattersall et al. 2018). Thermal windows are also found on the body trunk of seals and may facilitate heat dissipation and evaporation of water trapped within the wet pelage (Mauck et al. 2003).

Similar to the appendages of marine mammals, the penguin flipper is a large, relatively uninsulated structure that might serve as a thermal window available for control of heat transfer via vasomotor action and countercurrent heat exchange (Frost et al. 1975; Boyd and Croxall 1996; Thomas 2007; Thomas and Fordyce 2012). This may lead to a large temperature gradient between the distal and proximal flipper in some situations (e.g., during extended periods at sea and during dives) (Prévost and Sapin-Jaloustre 1964; Ponganis et al. 2003), and a uniformly heated flipper that is maintained well above ambient temperature in others (e.g., during rest on land and at the sea surface) (Ponganis et al. 2003; Schmidt 2006). Uninsulated legs and feet of other seabirds have been identified as analogous heat-transfer structures (Irving and Krog 1955; Kilgore and Schmidt-Nielsen 1975; Baudinette et al. 1976) with countercurrent heat exchangers and main blood vessels with sphincteric action (Johansen and Millard 1973; Hargens et al. 1978; Midtgård 1981; see Johansen and Bech 1983 for review) that allows fine-scale control of leg and foot temperature (Kazas et al. 2017).

The aim of this study was to describe patterns of surface rewarming in plausible thermal windows (flippers, feet) in king penguins after their return to land following foraging bouts at sea. We also studied the simultaneous rewarming of surface temperature on the well-insulated body trunk, to gain insights into the relative roles of uninsulated but relatively small, and well insulated, considerably larger, surface areas in the control of heat transfer (McCafferty et al. 2013). Using thermal imaging, we were able to collect rewarming data more closely linked to heat exchange patterns, which is arguably relevant for the study of thermal windows, compared to previous work on rewarming in penguins that used internally implanted sensors (Ponganis et al. 2003; Schmidt 2006). We specifically studied how surface temperature recovery was affected by internal temperature, testing the hypothesis that birds facilitate the recovery of internal temperature by delaying peripheral temperature recovery. This was achieved by performing an experiment where we compared the dynamics of rewarming of king penguins that had just returned from the sea. These individuals showed both low internal and low peripheral temperature (henceforth ‘fully hypothermic’; i.e., all tissue temperatures ≥ 2 °C below normothermia), similar to the physiological state of penguins when feeding (Handrich et al. 1997). We then measured the same birds once they had completed recovery (i.e., when internal temperature was ≥ 37 °C and peripheral temperature was stable) and we had induced peripheral hypothermia by cold water immersion (henceforth ‘partially hypothermic’). We predicted that we would observe a delay of surface rewarming in fully hypothermic birds compared to partially hypothermic birds, which could indicate that they reduced peripheral circulation until their internal temperature had reached normothermia, or simply reflect differences in heat transfer as the bird rewarms from the inside and out. Because the stress of handling can induce thermoregulatory changes in deep and peripheral tissues in both penguins and other birds (Regel and Pütz 1997; Herborn et al. 2015; Nord and Folkow 2019), we also compared rewarming patterns recorded in the laboratory with those in free-ranging, non-handled, birds in the colony after their return from sea. This study, therefore, provides insights into patterns of rewarming in hypothermic penguins as they transition from prolonged periods at sea to land, and how this process was affected by internal state.

## Materials and methods

**Fig. 1:**
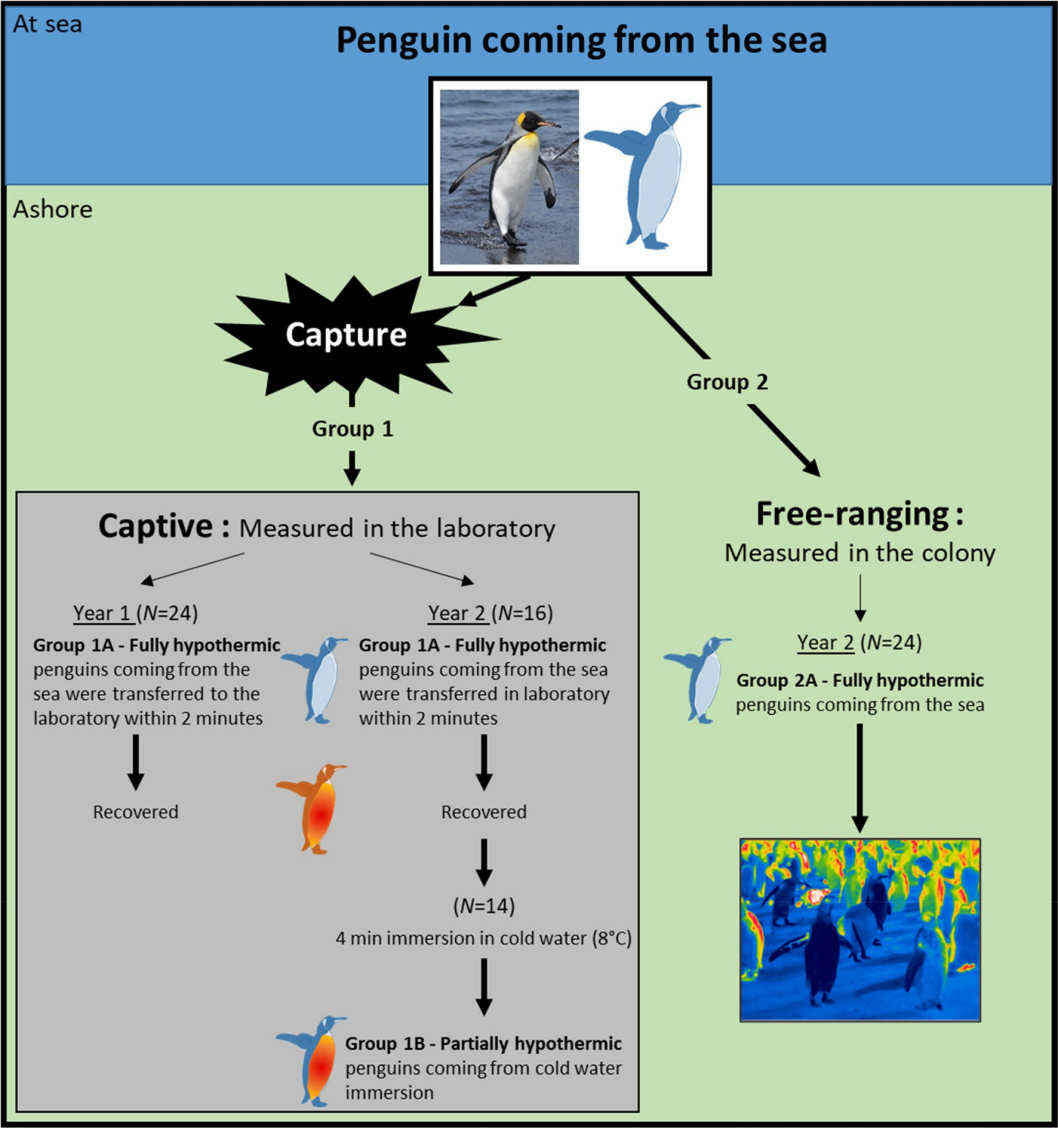
Graphic illustration of the experimental protocol used to investigate surface temperature rewarming ashore after a foraging trip in king penguins. Studies were performed on: (1) captive individuals that were measured in the laboratory after their return to the colony from a foraging trip at sea (Group 1); and (2) free-ranging birds that rewarmed in the colony after coming from the sea (Group 2). The birds in Group 1 were measured in two experimental conditions; once in a fully hypothermic state (i.e., directly when they came from the sea; 1A) and again in an experimentally induced partially hypothermic state (1B). The birds in Group 1 in Year 1, and all birds in Group 2, were measured only once. No birds were re-used between years or in the different groups

King penguins were studied during the courtship phase of their breeding cycle in La Baie du Marin on Possession Island, Crozet Archipelago ((46°4′ S, 51°8′ E) from November to March during two consecutive summers (Year 1: 2015–2016; Year 2: 2016–2017). The experimental design is outlined in Fig. 1.

### Effects of internal temperature on surface rewarming—Group 1

Thermal images of individual king penguins (*N* = 40; Year 1: 24; Year 2: 16) were collected using a thermal imaging camera (ThermaCAM™ P25, FLIR Systems, Orsonville, Florida, USA, accuracy ± 2 °C) to confirm that birds were in a peripherally vasoconstricted state. This was clearly visible as uniformly cold body trunk, head, beak and flippers (Fig. 1). The bird was then captured and transferred to a thermal imaging studio in a nearby laboratory within 2 min (Year 1: 2.07 min ± 0.10 s; Year 2: 2.53 min ± 0.42 s, mean ± s.e.) (Group 1A, Fig. 1). The thermal imaging studio (hereafter referred to as the ‘laboratory’) provided standardized conditions for thermal imaging by excluding wind, precipitation and solar radiation, and also allowed for a uniform angle and distance (1.1 m) of measurement. We fitted birds with a hood before measurements to reduce stress (Cockrem et al. 2008). We then proceeded to collect thermal images of the ventral surface of the right flipper (*T*_flipper_) and breast (*T*_breast_) just below the axilla. Because the flipper had to be manually opened to expose the ventral side, we only measured rewarming in one flipper to reduce handling stress. We also collected a thermal image of the dorsal area of the right foot (*T*_foot_). This set of two images, i.e., flipper/breast and foot, was collected every 30 s. In Year 1, internal temperature was not measured, but in Year 2, it was measured by inserting a digital thermometer with a flexible tip (Gilbert, Caen, FR) 6–8 cm through the cloaca (*T*_cloacal_). Data in Year 1 were collected for 6.4 min ± 0.5 min (s.e.) after capture (range 6–12 min). To ascertain that birds had sufficient time to reach a stable surface temperature during the experiment, we extended measurement duration to 21.3 min ± 0.5 min in Year 2 (range 16.9–32.4 min). Activity during the measurements were scored on a scale from 1 to 3, where 1 represented a calm individual, and 3, a distressed individual that continuously attempted to escape. Activity level did not affect any measured temperatures (all *P* > 0.08), and some birds even appeared to be asleep during measurements (AL, pers. obs.). This effect was, therefore, not considered further below. Mean air temperature, *T*_a_, in the laboratory was 8.1 ± 1.4 °C (s.d.) during all trials (range 4.7–11.4 °C), which is within the thermoneutral zone of king penguins (i.e.,  − 5 to + 15 °C; Le Maho and Despin 1976; Froget et al. 2002; Fahlman et al. 2004).

Of the 16 individuals measured when fully hypothermic in Year 2, 14 were subsequently kept in thermoneutrality in an outdoor roofless wooden enclosure (3 × 3 m) without human disturbance for 68.00 ± 3.00 min to allow rewarming to internal normothermia. These birds were then brought back to the laboratory and thermal images of the flipper and the foot were taken, and *T*_cloacal_ (which was 2.2 ± 0.7 °C greater than when the birds had just returned from sea) was recorded. We then briefly immersed all of the body but the head in cold water (ca. 8 °C) that was available from a freshwater supply at the field site. The water bath had similar temperature to seawater in the adjacent bay (7.7 ± 0.9 °C in the same year; Lewden et al. 2017b). Immersion lasted 4.05 min ± 0.25 s, which was enough to induce peripheral vasoconstriction without changing internal temperature. Accordingly, *T*_cloacal_ did not differ before (37.3 ± 0.3 °C) and after immersion (37.2 ± 0.2 °C) (paired *t* test; df = 11 *t* =  − 0.33, *P* = 0.75), but we observed a significant decrease in *T*_flipper_ (before: 16.2 ± 1.4 °C; after: 11.0 ± 0.5 °C) (df = 13 *t* =  − 3.22, *P* = 0.006), *T*_foot_ (before: 15.8 ± 2.7 °C; after: 10.7 ± 0.5 °C) (df = 8, *t* =  − 0.99, *P* = 0.035) and *T*_breast_ (before = 15.5 ± 0.8 °C; after = 12.9 ± 0.5 °C) (df = 13 *t* =  − 2.77, *P* = 0.0101). Birds were then directly returned to the laboratory to be measured a second time (Group 1B, Fig. 1) during 21.0 min ± 0.1 min (range 17.3–25.3 min). The birds were then released in the colony.

### Measurements in the colony–Group 2

Thermal imaging of free-ranging birds (*N* = 24, Fig. 1), which had not been previously measured in the laboratory, was undertaken during Year 2, by following individuals from the point at which they returned from the sea for as long as possible before they could not be seen by the observer (Fig. 1, Free-ranging–Group 2). Data were collected on days without rain for, on average, 14.53 min ± 2.05 min (range 2.77–38.93 min). Images were collected as frequently as possible when birds were in view and the inside of the flipper was visible. This provided, on average (± s.e.), 22 ± 3 thermal images per bird, collected roughly every 1.6 min. Surface temperature were extracted only from thermal images where the regions of interest where displayed as in the laboratory. *T*_a_ within the colony was recorded every 3 min with an iButton (MXMDS1921Z-F5; Maxim Integrated, San Jose, CA, USA). We also scored wind speed from low (0) to high (4) and estimated percentage cloud cover. *T*_a_ ranged from 4.8 to 9.9 °C (mean: 7.4 ± 0.1 °C), cloud cover between 30 and 90% (mean: 69 ± 4%), and mean wind speed score was 1.70.

### Thermal image analysis

Mean *T*_flipper_ and *T*_foot_ were delineated by fitting polygons around each region of interest with the software ThermaCAM™ Researcher Pro 2.10 (Flir systems, Wilsonville, Oregon, USA). Emissivity was set to 0.98. Mean *T*_breast_ was extracted from a region just below the axilla, using a standardized square with length of side equal to the height of the distal flipper. We adjusted all images for variation in *T*_a_ and measurement distance (which was always 1.1. m in the laboratory). For field measurements, distance was not measured in meters and, therefore, we derived a distance index by counting the number of pixels along a line fitted from the top of the head to base of the foot. A greater number of pixels indicated that an individual was closer to the camera.

### Statistical analysis

For laboratory data, general linear mixed models (GLMMs) were used to model the initial temperature and the temperature change (i.e., final temperature–initial temperature; Year 2) for *T*_cloacal_, *T*_flipper_, *T*_foot_, and *T*_breast_, using thermal state (fully or partially hypothermic) and ambient temperature (*T*_a_) as explanatory variables, and bird ID as a random factor. For these analyses, only data recorded during Year 2 were used, as birds were not measured in both states in Year 1 (above) and we were specifically interested in comparing temperature change within individuals.

For data collected in the colony, we used GLMMs to model separately *T*_flipper_, *T*_foot_, and *T*_breast_ recorded as a function of time on land, distance (as indexed by pixel number; see above), and *T*_a_, wind index and cloud cover. Bird ID was used as a random factor to account for repeated measurements.

To compare rewarming patterns in the laboratory and in the colony, we first divided temperature data (for each tissue) into 2.5 min intervals between 0 ≤ *t* ≤ 5 min, and in 5 min intervals from 5 < *t* ≤ 25 min. To test if there was a delay, which we defined as the time necessary to measure a significant temperature increase compared to initial values, we then compared time-wise temperature differences for flippers, feet and breast between fully hypothermic birds in the laboratory (Year 1 and 2), partially hypothermic birds in the laboratory (Year 2), and free-ranging birds in the colony (Year 2) using GLMMs including *T*_a_, time interval, year nested in thermal state, the interaction between time interval and thermal state (nested in year), and bird ID as random factor. *T*_cloacal_, that was measured only in Year 2 in the laboratory, was compared between fully and partially hypothermic birds using a GLMM with *T*_a_, time interval and thermal state, and the interaction between time interval and thermal state, as explanatory variables and bird ID as random factor.

Statistical analyses were performed using JMP® v. 13 (SAS Institute Inc., Cary, North Carolina, USA). Results are presented as mean ± s.e. with capital “*N*” corresponding to the number of individuals and lowercase “*n*” to the total number of measurements. Non-significant parameters were excluded by backward elimination (Quinn and Keough 2002). A Student’s *t* test was used to compare the significant difference between two groups, whereas a Tukey HSD post hoc was used to compare three, or more, groups.

## Results

### Effects of internal temperature on surface rewarming—Group 1

**Fig. 2:**
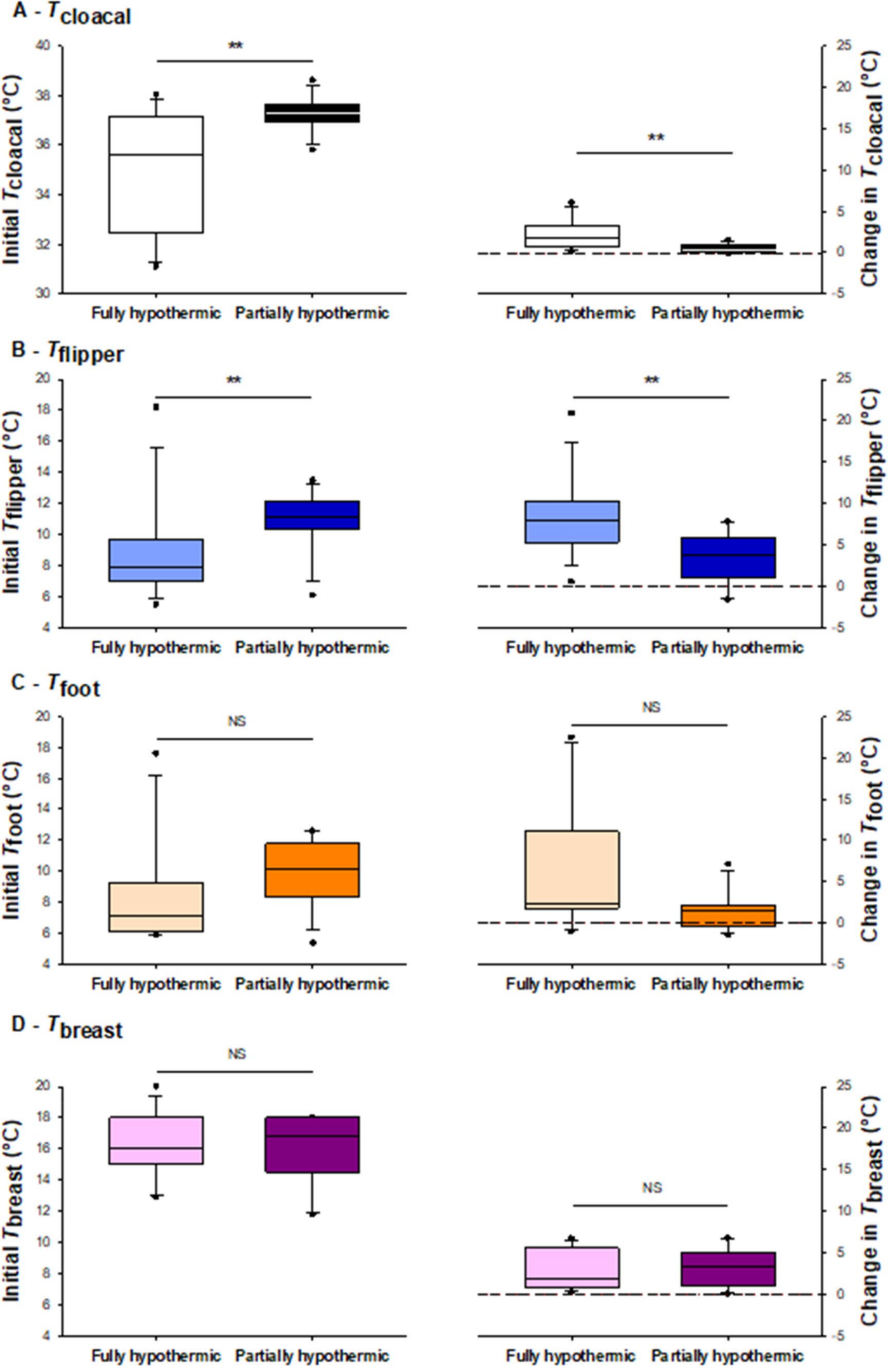
Differences in initial temperature (left) and temperature change (i.e., final temperature–initial temperature) (right) in hypothermic (dark boxes) and normothermic (light boxes) birds measured in the second year of the study. The panels show: cloacal temperature (*T*_cloacal_; **a**), flipper surface temperature (*T*_flipper_; **b**), foot surface temperature (*T*_foot_; **c**) and breast surface temperature (*T*_breast_; **d**). Boxes show medians (white and black lines), and 1st and 3rd quartiles (upper and lower margins of the box). The whiskers extend to the 5th and 95th percentiles. Significance was assessed using a Student’s *t* test post-hoc. The dashed line denotes no change in tissue temperatures. *NS* non-significant, **P ≤ 0.047

In laboratory conditions during Year 2, all tissues reached a high stable temperature at the end of the experiment (mean of 37.3 ± 0.3 °C; 15.9 ± 0.8 °C; 12.5 ± 1.1 °C; and 19.1 ± 0.3 °C, respectively, in the cloacal, flipper, foot and breast areas). However, due to lower initial temperature in fully compared to partially hypothermic birds (Fig. 2), the measured body temperature change was greater in the fully hypothermic birds (Fig. 2).

Fully hypothermic birds had lower initial *T*_cloacal_ (35.0 ± 0.5 °C) than partially hypothermic individuals (36.9 ± 0.5 °C) (*P* = 0.007, *N* = 14) (Fig. 2a), with a positive effect of *T*_a_ (*P* = 0.047, *N* = 28). There was no effect of *T*_a_ on the change in *T*_cloacal_, or on the change in any of the other measured temperatures. Initial *T*_flipper_ was also lower in fully hypothermic (8.9 ± 0.8 °C) than in partially hypothermic birds (12.3 ± 1.0 °C) (*P* = 0.012; Fig. 2b), but the difference in *T*_foot_ between treatments only approached statistical significance (fully hypothemic: 8.3 ± 0.9 °C; partially hypothermic: 10.8 ± 0.8 °C; *P* = 0.063; Fig. 2c). There was no treatment-wise difference in initial *T*_breast_ (fully hypothermic: 16.2 ± 0.6 °C; partially hypothermic: 16.0 ± 0.6 °C; *P* = 0.8; Fig. 2d).

**Fig. 3:**
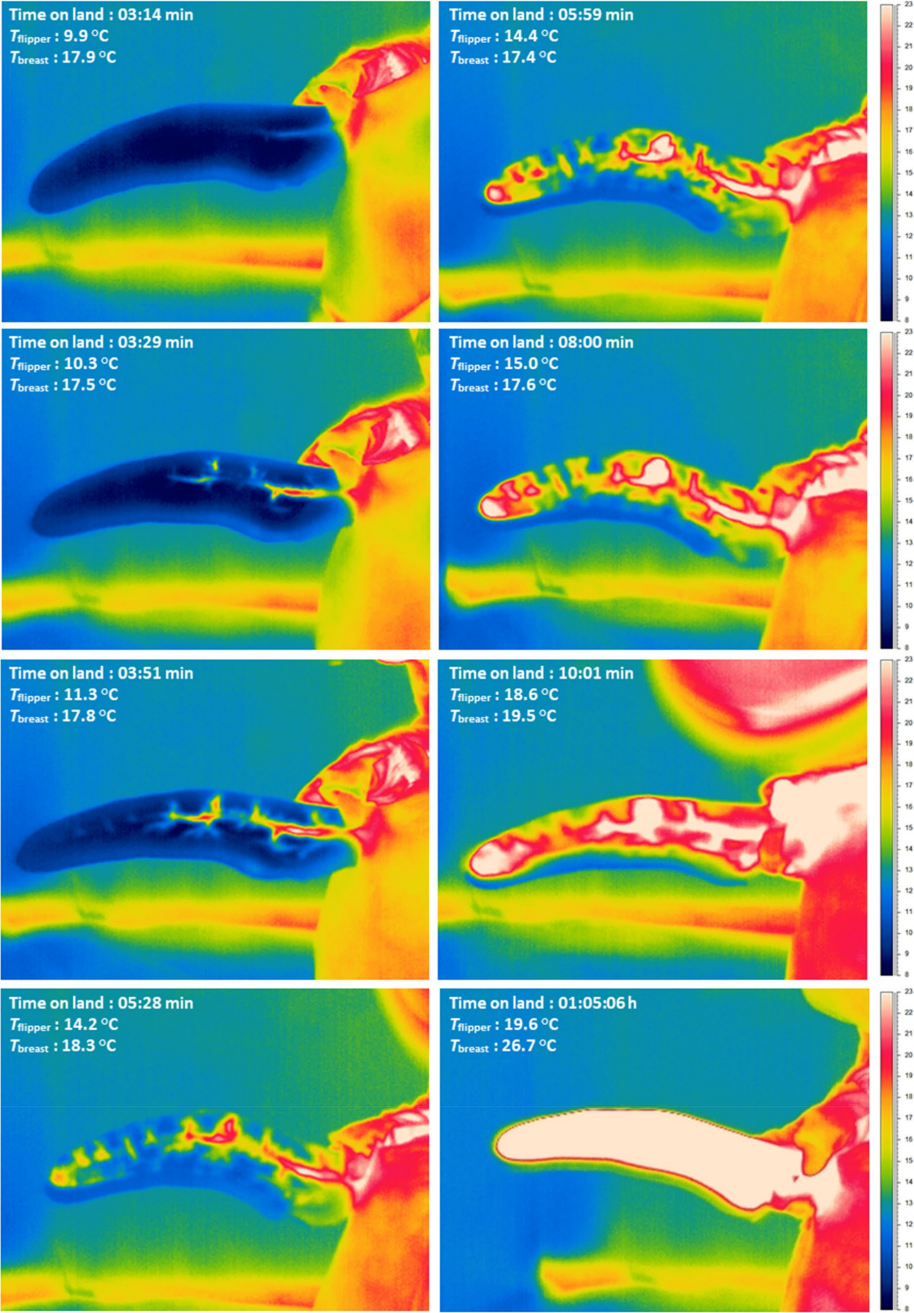
Representative set of thermal images showing the rewarming of flipper and breast surface temperature in a fully hypothermic bird (ID 9, Group 1A) measured in the laboratory after its return from the sea. Time on land (min) and mean *T*_flipper_ and *T*_breast_ are shown in each panel

*T*_cloacal_ increased over time in the fully hypothermic group (+ 2.1 ± 0.5 °C), but was stable in the partially hypothermic treatment (+ 0.5 ± 0.1 °C) (*P* = 0.004; Fig. 2a; Table 1). *T*_flipper_ increased within the first 10 min of measurement in the fully hypothermic birds (Fig. 2b), e.g., an increase from 9.9 to 18.6 °C in penguin ID9 (Fig. 3). At the end of the 21 min study period, the change in *T*_flipper_ was greater in fully hypothermic (+ 8.3 ± 1.4 °C) than in partially hypothermic (+ 3.3 ± 0.9 °C) birds (*P* = 0.002; Fig. 2b). The positive change in *T*_foot_ in fully hypothermic birds showed a slight tendency to be more pronounced (+ 6.2 ± 2.4 °C) compared to the partially hypothermic penguins (+ 1.5 ± 0.7 °C) (*P* = 0.07; Fig. 2c). Mean *T*_breast_ also increased during the first 10 min of measurement, e.g. from 17.9 to 19.5 °C in penguin ID9 (Fig. 3). The change in *T*_breast_ (i.e., final temperature–initial temperature) was similar in fully hypothermic (+ 2.9 ± 0.6 °C) and partially hypothermic (+ 3.2 ± 0.6 °C) states (*P* = 0.88; Fig. 2d).

**Table 1:**
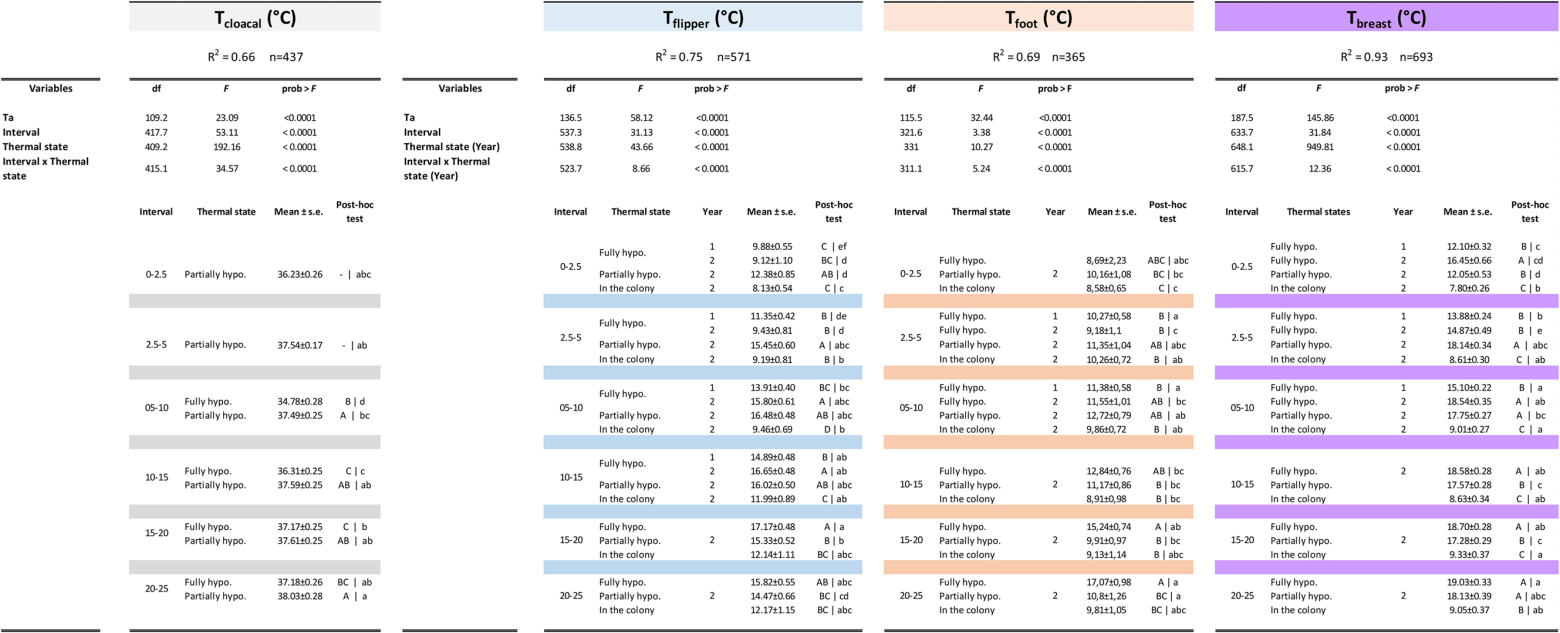
Final models of temperature recovery as a function of thermal states in king penguins that rewarmed either in the laboratory (fully and partially hypothermic) or in the colony

Data were analysed using general linear mixed effects models with bird ID as a random intercept, with separate models for each tissue. Post hoc tests were performed both between time intervals within thermal state (capital letters to the left of the vertical line) and or the thermal states within time intervals (lowercase letters to the right of the vertical line). Different letters denote significant (*P* < 0.05) differences

### Measurements in the colony—Group 2

In the colony, we observed a linear increase of *T*_flipper_, *T*_foot_ and *T*_breast_ with time on land (Table 2; all *P* < 0.001). Moreover, *T*_foot_ and *T*_breast_, but not *T*_flipper_ increased with increasing *T*_a_ (Table 2). We also noted that *T*_flipper_, but not *T*_foot_ and *T*_breast_, increased as measurements were closer (Table 2). There was no effect of cloud cover on any surface temperature (*P *> 0.1), but we observed a significant negative relationship between *T*_flipper_ and wind index (Table 2).

**Table 2:**
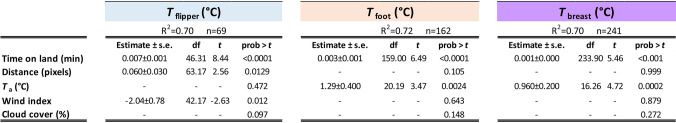
Effects of time on land, distance, *T*_a_, wind index and cloud cover on *T*_flipper_, *T*_foot_ and *T*_breast_ surface temperatures in free-ranging king penguins measured in the colony starting immediately upon their return to land after a foraging trip at sea

Data were analysed using general linear mixed effects models with bird ID as a random intercept, with separate models for each tissue

### Comparison of rewarming in the laboratory and in the colony

The increase in tissue temperature with time differed between thermal states (i.e., interval × thermal state: *P* < 0.0001) (Table 1). There was also a significant positive effect of *T*_a_ (*p* < 0.0001), and a strong positive effect of time interval (*P* < 0.0001) on all tissue temperatures and in all thermal states.

### Recovery patterns within individual thermal states

**Fig. 4:**
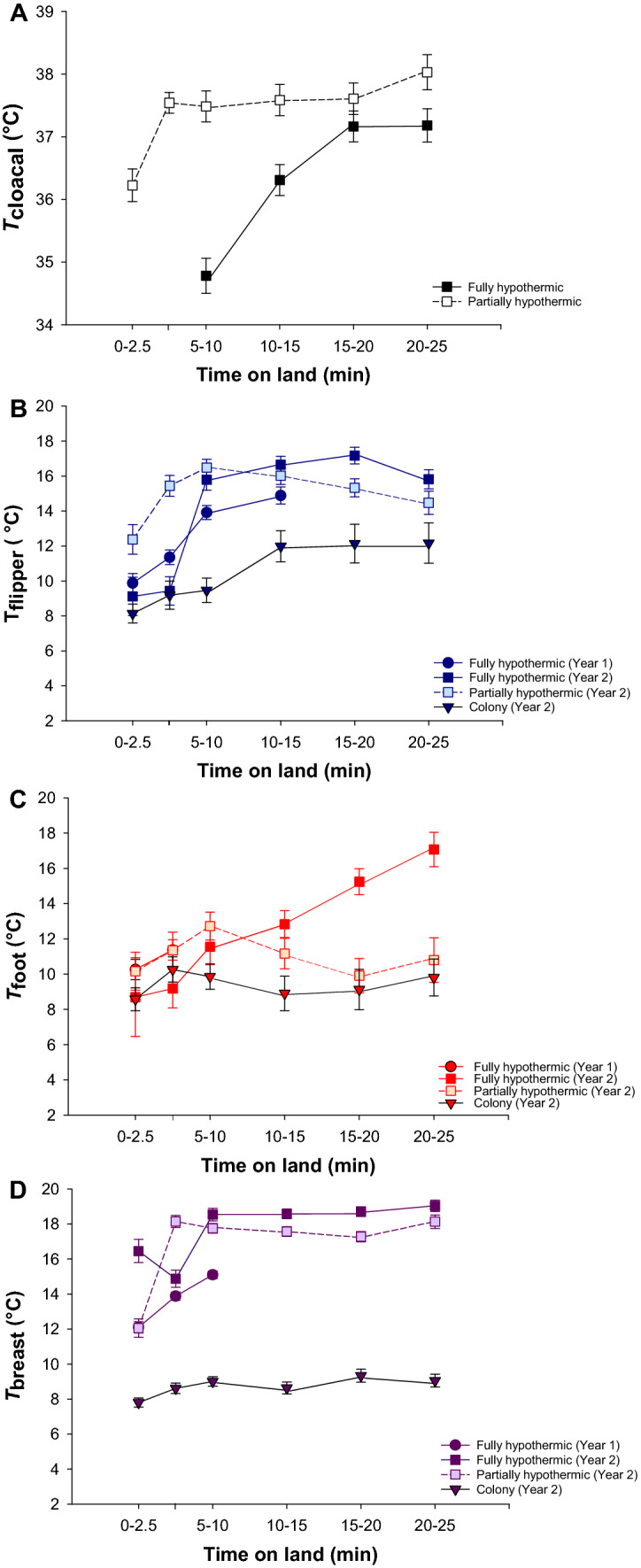
Mean (± s.e.) *T*_cloacal_ (**a**), *T*_flipper_ (**b**), *T*_foot_ (**c**) and *T*_breast_ (**d**), as a function of time on land in fully hypothermic (Year 1: filled circles and solid line; Year 2: filled squares and solid line), partially hypothermic (light squares and dashed line) and free-ranging birds (filled triangles and solid line). Mean temperatures were calculated by averaging temperatures measured between 00:00 and 2:30 min for the first interval, between 2:31 and 5:00 min for the second interval, between 5:01 and 10:00 min for the third interval, and so on. Results from post hoc comparisons between intevals within thermal states, and between thermal states within intervals, are presented in Table 2

In partially hypothermic birds, *T*_cloacal_ maintained a stable normothermic value (38.0 ± 0.3 °C) with the 21 min measurement period (Table 1). The fully hypothermic birds also recovered *T*_cloacal_, but this increase was delayed compared to the partially hypothermic birds (Fig. 2a). However, since we recorded the first *T*_cloacal_ after 5 min (see Methods), we cannot fully compare the recovery pattern at the start of rewarming (Fig. 4a).

*T*_flipper_ in fully hypothermic birds did not increase within the first 5 min on land (Fig. 4b). In partially hypothermic birds, *T*_flipper_ started to increased immediately, such that it was higher than in the fully hypothermic group after 5 min (Time interval 2.5–5 Table 1; Fig. 4b). *T*_flipper_ in the colony increased only slightly, but non-significantly so, during the observation period (Fig. 4b).

*T*_foot_ increased gradually in fully hypothermic birds (Fig. 4c). However, we did not observe any significant change in partially hypothermic birds measured in the laboratory, or in birds measured in the colony (Table 1; Fig. 4c).

*T*_breast_ started to increase above baseline values only after 5 min on land (Fig. 4d). However, the increase was immediate in the partially hypothermic group, such that *T*_breast_ was higher than in the fully hypothermic grouped after 2.5 min (Table 1). *T*_breast_ then remained stable until the end of experiment in both groups (Fig. 4d). There was globally no change in *T*_breast_ over the observation period in the colony (Table 1; Fig. 4d).

### Time-wise differences in recovery patterns between thermal states

Fully hypothermic birds showed a significant lower initial *T*_flipper_ until 5 min (i.e., a delay of 5 min) compared to the partially hypothermic group and penguins measured in the colony, with no difference between the latter two groups (Table 1; Fig. 4b). Moreover, we noted that at the end of the recovery, the final *T*_flipper_ was similar in all thermal states (Time interval 20–25 Table 1; Fig. 4b).

*T*_foot_ in penguins during recovery showed a similar pattern during the first 10 min in all years and thermal states (Table 1; Fig. 4c). The subsequent increase in *T*_foot_ in fully hypothermic birds (Fig. 4c) resulted in a higher *T*_foot_ than in the partially hypothermic group and penguins measured in the colony (Time intervals 15–20 and 20–25 Table 1; Fig. 4c).

*T*_breast_ recorded in the laboratory was constantly higher than in the colony (Fig. 4d). Initial *T*_breast_ was lower in laboratory during Year 1 than during Year 2 (Time interval 0–2.5 Table 1; Fig. 4d), but this difference disappeared after 2.5 min (Table 1). There was no difference in *T*_breast_ recovery in fully and partially hypothermic birds (Time interval 20–25 Table 1; Fig. 4d).

## Discussion

Our study revealed rapid vasodilation of the king penguin flipper after the return from the sea, though this was only observed when viewed at close proximity when the birds were allowed to recover from their hypothermic state indoors sheltered from the environment in the laboratory (Figs. 2b and 3). Rewarming of *T*_breast_ followed a similar trajectory, although the thicker insulation covering this part of the body meant we could not image any vasodilation response (Fig. 3). Rewarming of *T*_foot_ showed a more ambiguous pattern over our observation period, tending to increase only in the fully hypothermic laboratory birds (Figs. 2c and 4c). Our measurement of lower post-recovery *T*_flipper_ compared to previous studies of king and emperor penguins (*Aptenodytes forsteri*) (Ponganis et al. 2003; Schmidt 2006) probably reflects the fact that we measured a wholly external temperature that was integrated over the full surface area of the flipper. This temperature is expected to be lower than the recordings from more proximally positioned, internal, sensors (Ponganis et al. 2003; Schmidt 2006). In line with this, maximum *T*_flipper_ (26.7 ± 0.23 °C; Fig. S1) was well below internal flipper temperatures in previous studies (35–38 °C in Ponganis et al. 2003; around 38 °C in Schmidt 2006).

### Effects of internal temperature on surface rewarming

Fully hypothermic birds (i.e., Group 1A), showed relatively large surface temperature changes during measurements (Figs. 2 and 4b–d). During the same time period, cloacal temperature also increased and had attained a stable value well before the end of measurements (Fig. 4a). In contrast, partially hypothermic birds showed a more moderate increase in surface temperature during the same experiment duration, likely because of the higher initial flipper temperature (Figs. 2b and 4b), with only minor changes in foot temperature (Figs. 2c and 4c). Cloacal temperature also increased in the partially hypothermic birds from the first measurement onwards (Fig. 2a), and had reached normothermia before the end of the observation period (Fig. 4a). This confirms that, in king penguins, rewarming of peripheral structures does not prevent recovery of internal temperature. However, according to our prediction, the fact that both flipper and breast temperature started to increase after a delay of 5 min in the fully hypothermic birds (Fig. 4b and d), suggests that low internal temperature modifies the peripheral recovery pattern, most likely because more heat needs to be produced before normothermia is reached. It could also be due to fact that lower internal tissue temperature delays peripheral rewarming when centrally produced heat is transferred through the body before reaching the appendages and body surface. Alternatively, this could indicate that preference is given to reducing heat loss to restore core temperature, but in that case, we probably would not expect a similar onset of rewarming in the two groups of birds.

Some birds maintained vasoconstriction in the flippers and feet during the observation period (Fig. S1). This flexibility of vasomotor states in the appendages, and their superficial vasculature (e.g., Fig. 3), highlight their roles as potential thermal windows. In contrast to studies on seals (Mauck et al. 2003), we did not observe any indication of similar thermal windows over the body trunk. This is expected, because the thick plumage that provides up to 80% of insulation in penguins (Le Maho et al. 1976; Le Maho 1977) would leave little room for any meaningful circulatory adjustment of local body trunk temperature. Because we could not image the underlying vasculature, we also do not know if the observed increase in *T*_breast_ reflected peripheral vasodilation, increased heat production in the underlying pectoral muscles (e.g., Hohtola 2012), or largely a consequence of drying plumage that would improve insulation and decrease evaporative cooling and, hence, reduce heat loss (De Vries and van Eerden 1995). It would be interesting to investigate this potential effect of changes to evaporation during recovery in future studies. Regardless of the mechanism involved, the high absolute *T*_breast_ combined with the considerably larger surface area of this part of the body compared to that of the appendages, means that the body trunk makes a major contribution to overall heat exchange with the environment, as is also known in the related emperor penguin (McCafferty et al. 2013).

### Comparison of rewarming in the laboratory and in the colony

Free-ranging birds in the colony showed very slight rewarming of peripheral regions (Fig. 4), which contrasts with the laboratory results. We do not believe that the different rewarming patterns in the laboratory were entirely a consequence of a thermoregulatory response to capture and handling. For example, if increased *T*_cloacal_ was caused by stress from repeated handling in the laboratory (e.g., Cabanac and Guillemette 2001), we would not have expected this temperature to stabilize in fully hypothermic birds by the end of the observation period and then to remain relatively stable until the start of the second trial (Fig. 4a). This response was qualitatively similar to that recorded by abdominally implanted temperature sensors in king penguins that were handled in the same way but then left to recover to normothermia in an unrestrained state without human disturbance during 2 h (Lewden et al. *in press*). There was also no further change in *T*_flipper_ for about 1 h after the end of handling, neither in birds that rewarmed from hypothermia nor in birds that were captured and handled in a similar way when they were already completely dry and in a presumed normothermic state in the colony (Fig. S2). Had the initial increase in *T*_flipper_ indeed been a thermoregulatory response to reduce core temperature after the acute stress response (Briese and Cabanac 1991; Cabanac and Guillemette 2001), we may have expected reversal to a lower baseline starting at the end of handling (Herborn et al. 2015). Finally, there was no effect of activity score on rewarming patterns (see “Materials and methods” section), and we observed that several individuals fell asleep during measurements. This suggests that stress-induced activity metabolism was not obvious. Hence, we do not believe that the recovery pattern measured in the laboratory was driven by the stress of capture, even though it could explain some difference between free-ranging and laboratory birds.

Differences between the groups could instead be attributed to differences in the physical environment between the laboratory and the field, manifested e.g. in faster drying of the plumage in the laboratory and consequent higher initial *T*_breast_ compared to in the colony (Fig. 4d). In line with this, the non-insulated body parts (flippers and feet) showed no difference in initial temperature between the two conditions (Fig. 4b and c). If variation in the physical environment indeed increased the overall rate of heat transfer in the colony (cf. Gates 1980; Monteith and Unsworth 2013), it is possible that the lack of peripheral rewarming when birds were measured in the free-ranging condition is consistent with vasoconstriction of peripheral and/or poorly insulated structures to avoid excessive heat loss until internal temperature has recovered. To elucidate this possibility, it would be interesting to compare the recovery rates of core and subcutaneous temperatures in free-ranging birds with implanted loggers. Future studies should also aim to extend our experimental protocol by studying rewarming rates in fully hypothermic and partially hypothermic penguins that are allowed to recover in a range of controlled environmental temperatures.

## Electronic supplementary material

Below is the link to the electronic supplementary material.

Supplementary file1 (DOCX 116 kb)

## References

[CR1] Baudinette RV, Loveridge JP, Wilson KJ, Mills CD, Schmidt-Nielsen KNUT (1976). Heat loss from feet of herring gulls at rest and during flight. Am J Physiol.

[CR2] Bevan RM, Boyd IL, Butler PJ, Reid K, Woakes AJ, Croxall JP (1997). Heart rates and abdominal temperatures of free-ranging South Georgian shags, *Phalacrocorax georgianus*. J Exp Biol.

[CR3] Boyd IL, Croxall JP (1996). Dive durations in pinnipeds and seabirds. Can J Zool.

[CR4] Boyd IL (2000). Skin temperatures during free-ranging swimming and diving in Antarctic fur seals. J Exp Biol.

[CR5] Bullard RW, Rapp GM (1970). Problems of body heat loss in water immersion. Aerospace Med.

[CR6] Briese E, Cabanac M (1991). Stress hyperthermia: physiological arguments that it is a fever. Physiol Behav.

[CR7] Cabanac AJ, Guillemette M (2001). Temperature and heart rate as stress indicators of handled common eider. Physiol Behav.

[CR8] Cockrem JF, Potter MA, Barrett DP, Candy EJ (2008). Corticosterone Responses to Capture and Restraint in Emperor and Adelie Penguins in Antarctica. Zoolog Sci.

[CR9] Croxall JP (1982). Energy costs of incubation and moult in petrels and penguins. J Anim Ecol.

[CR10] Culik BM, Pütz K, Wilson RP, Bost C-A, Le Maho Y, Verselin J-L (1996). Core temperature variability in diving king penguins (*Aptenodytes patagonicus*): a preliminary analysis. Polar Biol.

[CR11] De Vries J, van Eerden PW (1995). Thermal Conductance in aquatic bird in relation to the degree of Water Contact, Body Mass, and Body Fat : Energetic implications of living in a strong cooling environment. Physiol Zool.

[CR12] Erdsack N, Hanke FD, Dehnhardt G, Hanke W (2012). Control and amount of heat dissipation through thermal windows in harbor seals (*Phoca vitulina*). J Therm Biol.

[CR13] Fahlman A, Handrich Y, Woakes AJ, Bost C-A, Holder R, Duchamp C, Butler PJ (2004). Effect of fasting on the VO2-fh relationship in king penguins, *Aptenodytes patagonicus*. Am J Physiol Regul Integr Comp Physiol.

[CR14] Feltz ET, Fay FH (1966). Thermal requirements in vitro of epidermal cells from seals. Cryobiology.

[CR15] Froget G, Handrich Y, Le Maho Y, Rouanet J-L, Woakes AJ, Butler PJ (2002). The heart rate/oxygen consumption relationship during cold exposure of the king penguin: a comparison with that during exercise. J Exp Biol.

[CR16] Frost PGH, Siegfried WR, Greenwood PJ (1975). Arteriovenous heat- exchange systems in jackass penguin *Spheniscus demersus*. J Zool.

[CR17] Gates DM (1980). Biophysical Ecology.

[CR18] Hammond KA, Spotila JR, Standora EA (1988). Basking behavior of the turtle *Pseudemys scripta*: effects of digestive state, acclimation temperature, sex, and season. Physiol Zool.

[CR19] Handrich Y, Bevan RM, Charrassin J-B, Butler PJ, Putz K, Woakes AJ, Lage J, Le Maho Y (1997). Hypothermia in foraging king penguins. Nature.

[CR20] Hargens AR, Scholander PF, Orris WL (1978). Positive tissue fluid pressure in the feet of antarctic birds. Microvasc Res.

[CR21] Herborn KA, Graves JL, Jerem P, Evans NP, Nager R, McCafferty DJ, McKeegan DEF (2015). Skin temperature reveals the intensity of acute stress. Physiol Behav.

[CR22] Hohtola E, McCue MD (2012). Thermoregulatory adaptations to starvation in birds. Comparative Physiology of Fasting, Starvation, and Food Limitation.

[CR23] Irving L, Krog J (1955). Temperature of skin in the Arctic as a regulator of heat. J Appl Physiol.

[CR24] IUPS Thermal Commission (2003). Glossary of terms for thermal physiology. J Therm Biol.

[CR25] Johansen K, Bech C (1983). Heat conservation during cold exposure in birds (vasomotor and respiratory implications). Polar Res.

[CR26] Johansen K, Millard RW (1973). Vascular responses to temperature in the foot of the giant fulmar, Macronectes giganteus. J Comp Physiol A.

[CR27] Kazas S, Moran B, Saar G (2017). The Humboldt Penguin (*Spheniscus humboldti*) Rete Tibiotarsale - A supreme biological heat exchanger. J Therm Biol.

[CR28] Kilgore DL, Schmidt-Nielsen K (1975). Heat loss from ducks' feet immersed in cold water. Condor.

[CR29] Klir JJ, Heath JE (1992). An infrared thermographic study of surface temperature in relation to external thermal stress in three species of foxes: The Red fox (*Vulpes vulpes*), Arctic fox (*Alopex lagopus*), and Kit fox (*Vulpes macrotis*). Physiol Zool.

[CR30] Kvadsheim PH, Folkow LP (1997). Blubber and flipper heat transfer in harp seals. Acta Physiol Scand.

[CR31] Le Maho Y (1977). The emperor penguin: a strategy to live and breed in the cold. Am Sci.

[CR32] Le Maho Y, Delclitte P, Chatonnet J (1976). Thermoregulation in fasting emperor penguins under natural conditions. Am J Physiol.

[CR33] Le Maho Y, Despin B (1976). Réduction de la dépense énergétique au cours du jeûne chez le manchot royal *Aptenodytes patagonicus*. CR Acad Sci Paris D.

[CR34] Lewden A, Bonnet B, Nord N (2020). The metabolic cost of subcutaneous and abdominal rewarming in King Penguins after long-term immersion in cold water. J Therm Biol in press 10.1016/j.jtherbio.2020.102638 32716880

[CR35] Lewden A, Enstipp MR, Bonnet B, Bost C, Bost C-A, Handrich Y (2017). Thermoregulation strategy in fasting king penguin maintained in sea water tank. J Exp Biol.

[CR36] Lewden A, Enstipp MR, Picard B, van Walsum T, Handrich Y (2017). High peripheral temperatures in king penguins while resting at sea: thermoregulation versus fat deposition. J Exp BioL.

[CR37] Ling JK (1970). Pelage and molting in wild mammals with special reference to aquatic forms. Quarterly Review of Biology.

[CR38] Mauck B, Bilgmann K, Jones DD, Eysel U, Dehnhardt G (2003). Thermal windows on the trunk of hauled-out seals: hot spots for thermoregulatory evaporation?. J Exp Biol.

[CR39] Mccafferty DJ, Gilbert C, Thierry AM, Currie J, Le Maho Y, Ancel A (2013). Emperor penguin body surfaces cool below air temperature. Biol Let.

[CR40] Midtgård U (1981). The *Rete tibiotarsale* and arteriovenous association in the hind limb of birds: a compartive morphological study on counter-current heat exchange systems. Acta Zool.

[CR41] Monteith JL, Unsworth MH (2013). Principles of Environmental Physics.

[CR42] Nadel ER (1984). Energy exchanges in water. Undersea Biomed Res.

[CR43] Nord A, Folkow LP (2019) Ambient temperature effects on stress-induced hyperthermia in Svalbard ptarmigan. bioRxiv 10.1101/594614 10.1242/bio.043497 PMC6602330 31182628

[CR44] Norris AL, Houser DS, Crocker DE (2010). Environment and activity affect skin temperature in breeding adult male elephant seals (*Mirounga angustirostris*). J Exp Biol.

[CR45] Pabst DA, Rommel S, McLellan WA, Reynolds J, Rommel S (1999). The functional morphology of marine mammals. Biology of marine mammals.

[CR46] Ponganis PJ (2015). Diving Physiology of Marine Mammals and Seabirds.

[CR47] Ponganis PJ, Van Dam RP, Levenson DH, Knower T, Ponganis KV, Marshall G (2003). Regional heterothermy and conservation of core temperature in emperor penguins diving under sea ice. Comp Biochem Physiol A Mol Integr Physiol.

[CR48] Prévost J, Sapin-Jaloustre J (1964). A propos des premieres mesures de topographie thermique chez les Spheniscides de la Terre Adelie. Oiseau.

[CR49] Quinn GP, Keough JM (2002) Experimental Design and Data Analysis for Biologists. Cambridge University Press, New York

[CR50] Regel J, Pütz K (1997). Effect of human disturbance on body temperature and energy expenditure in penguins. Polar Biol.

[CR51] Ryg M, Lydersen C, Knutsen LO, Bjorge A, Smith TG, Øritsland NA (1993). Scaling of insulation in seals and whales. J Zool Lond.

[CR52] Schmidt A (2006) Etude de la thermoregulation en mer chez le manchot royal: Mecanismes et consequences energetiques - University of Strabourg. Ph.D thesis

[CR53] Schmidt A, Alard F, Handrich Y (2006). Changes in body temperature in king penguins at sea: the result of fine adjustments in peripheral heat loss?. Am J Physiol Regul Integr Comp Physiol.

[CR54] Scholander PF (1940). Experimental investigations on the respiratory function in diving mammals and birds. Hvalradets Skr.

[CR55] Tattersall GJ, Chaves JA, Danner RM (2018). Thermoregulatory windows in Darwin’s finches. Funct Ecol.

[CR56] Thomas DB (2007). The heterothermic loophole exploited by penguins. Aust J Zool.

[CR57] Thomas DB, Fordyce RE (2012). Biological plasticity in Penguin heat-retention structures. Anat Rec.

[CR58] Watts P (1992). Thermal constraints on hauling out by harbour seals (*Phoca vitulina*). Can J Zool.

[CR59] Watts P (1996). The diel hauling out cycle of harbour seals in an open marine environment: Correlates and constraints. J Zool Lond.

[CR60] Williams TM (1990). Heat transfer in elephants: thermal partitioning based on skin temperature profiles. J Zool Lond.

[CR61] Williams TM, Worthy GAJ, Hoelzel R (2002). Anatomy and physiology: the challenge of aquatic living. Marine Mammal Biology: an Evolutionary Approach.

