# Supplementary material for: Body surface rewarming in fully and partially hypothermic king penguins
Source: J Comp Physiol B. 2020 Jul 12;190(5):597–609. doi: 10.1007/s00360-020-01294-1 (PMC7441059; doi:10.1007/s00360-020-01294-1)
Supplement: Supplementary file 1 — Supplementary file1 (DOCX 116 kb) [file 360_2020_1294_MOESM1_ESM.docx]

**Supplementary material**


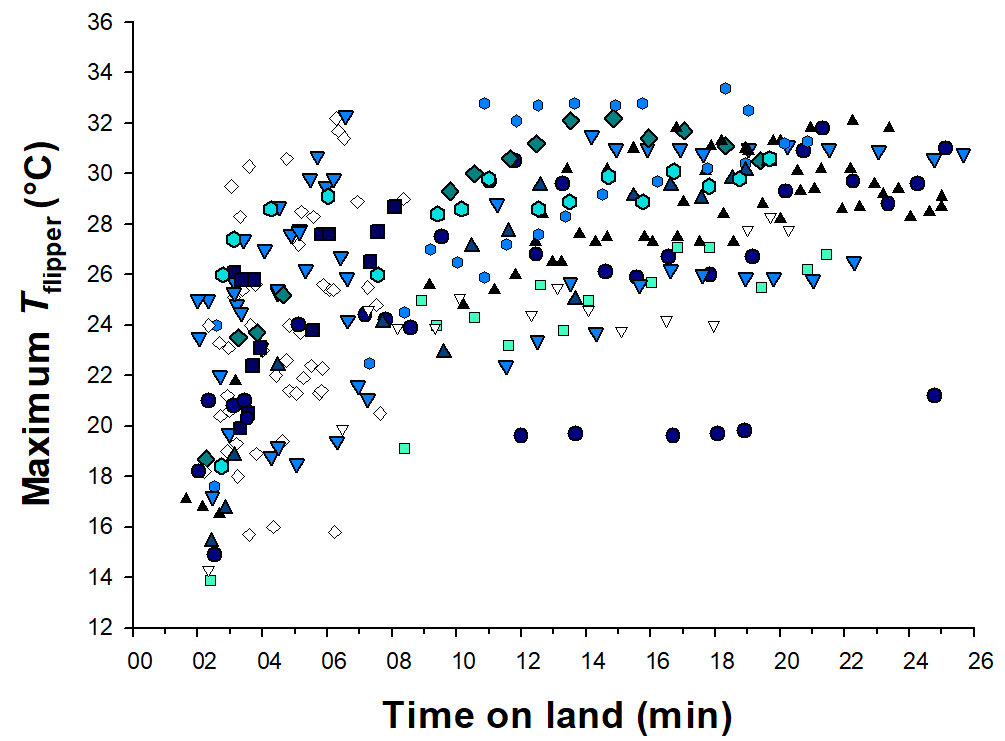


**Figure S1:** Maximum surface temperature in flippers from thermal images collected during rewarming in hypothermic king penguins measured in the laboratory. Each symbol corresponds to a different individual.


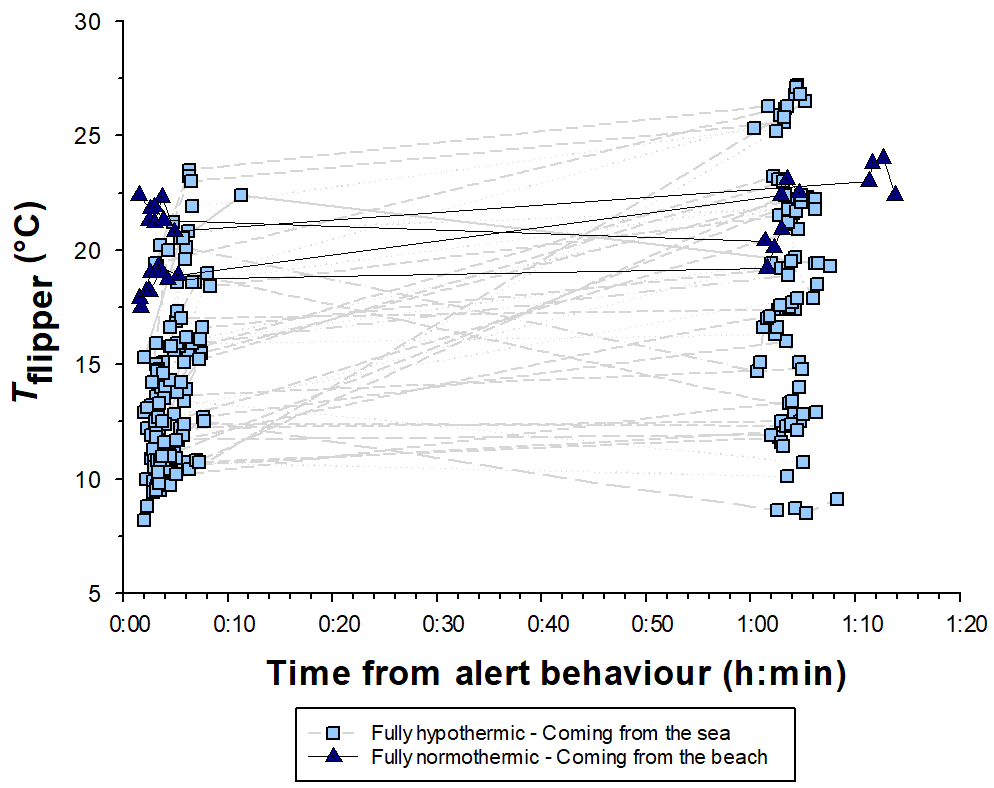


**Figure S2:** Change in flipper temperatures during rewarming in the laboratory in wet penguins caught just after their return from the sea (i.e., fully hypothermic - Light blue, *N*=24), and over the same time period in dry penguins caught when resting on the beach well after they had recovered from swimming-induced hypothermia (i.e., fully normothermic - Dark blue, *N*=4). Each line corresponds to a single individual.
